# Single-molecule measurements of bacteriophage lambda DNA packaging using purified terminase motor proteins and *E. coli* integration host factor

**DOI:** 10.1038/s41598-024-74915-2

**Published:** 2025-02-27

**Authors:** Brandon Rawson, Qin Yang, Carlos E. Catalano, Douglas E. Smith

**Affiliations:** 1 Department of Physics, University of California, San Diego, La Jolla, CA 92093 USA; 2 Department of Pharmaceutical Sciences, Skaggs School of Pharmacy and Pharmaceutical Sciences, University of Colorado Denver, Anschutz Medical Campus, Aurora, CO 80045 USA

**Keywords:** Molecular motor, Viral DNA packaging, Optical tweezers, Single-molecule biophysics, Terminase holoenzyme, Single-molecule biophysics, Phage biology

## Abstract

Biomotor-driven DNA packaging is a key step in the life cycle of many viruses. We previously developed single-molecule methods using optical tweezers to measure packaging dynamics of the bacteriophage lambda motor. The lambda system is more complex than others examined via single-molecule assays with respect to the packaging substrate and ancillary proteins required. Because of this, previous studies which efficiently detected packaging events used crude *E. coli* cell extracts containing host factors and the terminase packaging enzyme. However, use of extracts is suboptimal for biochemical manipulation and obfuscates interrogation of additional factors that affect the process. Here we describe an optical tweezers assay using purified lambda terminase holoenzyme. Packaging events are as efficient as with crude extracts, but only if purified *E. coli* integration host factor (IHF) is included in the motor assembly reactions. We find that the ATP-driven DNA translocation dynamics, motor force generation, and motor-DNA interactions without nucleotide are virtually identical to those measured with extracts. Thus, single-molecule packaging activity can be fully recapitulated in a minimal system containing only purified lambda procapsids, purified terminase, IHF, and ATP. This sets the stage for single-molecule studies to investigate additional phage proteins known to play essential roles in the packaging reaction.

## Introduction

**Fig. 1:**
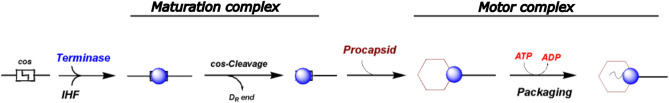
Schematic diagram of the phage lambda genome packaging pathway (see text for details). While details differ, the packaging pathway is strongly conserved amongst the large dsDNA viruses, both prokaryotic and eukaryotic.

Many dsDNA viruses use ATP powered molecular motors during assembly to package their genomes^1–3^. In most cases, a multi-subunit “terminase” motor complex assembles at a packaging initiation site within a multi-genome packaging substrate and cuts the duplex in preparation for packaging (see Fig. 1)^4–6^. This “maturation” complex then binds to a pre-assembled procapsid shell at a unique “portal vertex” to assemble a ring-shaped motor complex that translocates DNA into the procapsid, powered by ATP hydrolysis. DNA packaging is an energetically unfavorable process due to multiple factors including DNA self-repulsion, bending rigidity, entropy and hydration changes required to sequester the duplex into the confined capsid interior^7–10^. As a result, the molecular motor must exert significant force to reel the DNA into the capsid against high resisting forces.

Several phage and eukaryotic virus systems have been harnessed to study genetic, biochemical and structural features of the terminase packaging motors^1,5,11–16^. Three of these, phages phi29, T4, and lambda, have been adapted for biophysical interrogation via optical tweezers assays which can measure the packaging of single DNA molecules into single viral capsids in real time^17,18^. These studies have revealed general similarities, including the observation that these motors generate very high packaging forces of at least 50 pN, and they all translocate DNA with high processivity. Differences are also observed, however, in the DNA translocation velocities, velocity vs. capsid filling profiles, pausing and slipping events which occur during translocation, and strength of motor-DNA interactions measured in the absence of nucleotide^19–22^. This may reflect that the three phages have notable biological differences, such as in capsid shapes and sizes, genome lengths, and mechanisms of initiation and termination of packaging^5,6^.

The three model systems further represent three distinct packaging motor subtypes^23–25^. Phage phi29, a virus which infects *B. subtilis*, packages monomeric genomes and therefore does not employ a maturation complex. The motor is a pentameric complex that contains the packaging ATPase protein (gp16) and RNA molecules (pRNA)^2^. In contrast, phages T4 and lambda employ terminase motors as described above. The enzymes contain a large subunit (TerL), which harbors the nuclease (maturation) and translocase/ATPase activities, and a small subunit (TerS) which plays a role in the initiation of packaging. While T4 requires the TerS subunit in vivo, it is dispensable in vitro and optical tweezers studies have examined packaging by the isolated TerL subunit, in the absence of the TerS subunit^19^. Under these conditions, the T4 packaging reaction is non-specific and a variety of DNA substrates can be packaged^26^. In contrast, lambda terminase holoenzyme purifies as a stable complex of TerL and TerS subunits and DNA packaging measured in ensemble packaging reactions is strongly dependent on the *E. coli* integration host factor protein (IHF) in vivo and *in vitro*^27,28^. Moreover, packaging requires a DNA substrate that contains a *cos* sequence, the packaging initiation site, making packaging very specific (Fig. 1)^6^.

Optical tweezers studies of phage phi29 and T4 packaging dynamics have employed purified motor components, the packaging ATPase (gp16) and the large terminase subunit (gp17/TerL, in isolation), respectively. In contrast, lambda is the most complex of the three systems due to the in vitro requirement of a terminase holoenzyme composed of both gpA (TerL) *and* gpNu1 (TerS) subunits, the requirement of IHF and the requirement of a *cos*-containing packaging substrate^5,29^. Previous efforts in our lab to use purified lambda terminase proteins (and IHF) in the optical tweezers assay yielded some packaging events, but the efficiency of detecting events was very low relative to studies that employed crude cell extracts^17,19,30^ However, the use of crude extracts complicates reaction manipulation and detailed characterization of assembly and packaging conditions. Furthermore, the many host cell proteins/biomolecules present in extracts could exert effects on the process, either stimulatory or inhibitory, in a manner that would confound efforts to study the fundamental mechanisms of operation of the packaging motor. Indeed, bulk biochemical studies have demonstrated that IHF and nucleotides, and additional phage proteins such as gpFI can affect packaging reaction^5,31–33^. In the optical tweezers assay, components in the crude extracts could affect the efficiency and/or kinetics of initiation of packaging and/or details of the DNA translocation dynamics. Thus, it is desirable, as in many types of in vitro biochemical/biophysical studies, to identify the minimal system of components that can recapitulate the essential activity of interest^34^.

To address these issues, we here describe an optical tweezers protocol using highly purified lambda terminase holoenzyme (TerL·TerS_2_), that achieves packaging detection efficiencies comparable to our previous studies using crude cell extracts^19,22,30,35,36^. Consistent with in vivo and defined in vitro studies, we show that IHF is required to detect packaging events. Finally, we compare in detail the packaging dynamics and motor-DNA interactions measured using crude terminase from cell extracts with that measured using purified terminase holoenzyme. This study provides a minimal biochemical system in which to interrogate lambda genome packaging using single-molecule approaches and to define in detail the roles that ancillary proteins play in the packaging reaction.

## Materials and methods

### Preparation of the lambda terminase crude cell lysate

His-tagged lambda terminase holoenzyme was expressed in *E. coli* OR1265[pQH101] cells by heat induction and the cells were harvested by centrifugation as previously described^37^. The pellet was resuspended in 25 mM Tris–HCl, pH 8.6 at 4 °C, 2.5 mM MgCl_2_ and 10 mM DTT buffer and the cells were lysed by sonication. The lysate was centrifuged at 13,000×*g* for 10 min and glycerol was added to the clarified supernatant to a final concentration of 50%. This crude cell lysate was stored at − 20 °C.

### Purification of lambda terminase

The enzyme was purified previously described^37^, with modification. Briefly, lambda-terminase was expressed as above and the harvested cell pellets were taken into Buffer A (20 mM Tris–HCl, pH 8.6 at 4 °C, 500 mM NaCl, 10% (v/v) glycerol, 7 mM β-ME, 1 mM EDTA, and 20 mM imidazole). The cells were lysed by sonication and the lysate was clarified by centrifugation (13,000×*g* for 30 min). The clarified lysate was loaded onto a HisTrap FF column, and protein was eluted with a gradient to 500 mM imidazole in Buffer A. The terminase containing fractions were pooled and dialyzed against Buffer B (20 mM sodium phosphate, pH 6.8, 100 mM NaCl, 10% (v/v) glycerol, 7 mM β-ME, and 1 mM EDTA) to afford Pure 1 Terminase which was stored in 50% glycerol at − 80 °C. Next, the sample was loaded onto a HiTrap Q column and protein was eluted with a gradient to 1 M NaCl in Buffer B. Terminase containing fractions were pooled to afford Pure 2 Terminase which was stored which was stored in 50% glycerol at − 80 °C.

### Purification of integration host factor (IHF)

IHF was purified from HN880, a heat-inducible IHF overproducing strain as previously described^37^. Briefly, heat induced cells were harvested by centrifugation, and the pellet was taken into Buffer C (50 mM Tris–HCl buffer, pH 7.0, containing 20 mM KCl, 1 mM EDTA, 7 mM β-mercaptoethanol, 10% glycerol v/v) with protease inhibitor cocktail. The cells were lysed by sonication and the lysates were clarified by centrifugation (8000×*g* 30 min). Solid ammonium sulfate was added to the clarified supernatant to 45% saturation, and insoluble material was removed by centrifugation (8000*g* × 30 min). Ammonium sulfate was then added to 85% saturation, and the precipitated protein, which contained the majority of the IHF, was isolated by centrifugation (10,000*g* × 30 min). The pellet was taken into Buffer C and dialyzed against the same buffer overnight. The sample was loaded onto a HiTrap Q column (1 mL) equilibrated with Buffer C. The flow-through fraction, which contained IHF, was collected and then loaded onto a HiTrap Heparin HP (5 mL) column equilibrated with Buffer C. The protein was eluted with a linear gradient to 1.5 M KCl in Buffer C. IHF-containing fractions (∼ 870 mM KCl) were pooled, dialyzed against Buffer C containing 20% glycerol (v/v), and stored at − 20 °C.

### Purification of lambda procapsids

Procapsids were purified by published procedure^32^ except that the lambda lysogen MF869 (a generous gift of Dr. M. Feiss, U. Iowa) was used as the procapsid source. Briefly, heat induced cells were harvested by centrifugation and the pellet was resuspended in 50 ml ice-cold TMS buffer (50 mM Tris–HCl, pH 8.0, 10 mM MgCl_2_, 100 mM NaCl), the cells were lysed by sonication, and the crude lysate was clarified by centrifugation (8000×*g*, 10 min). The supernatant was spun at high-speed (130,000×*g*, 3 h), which pelleted the procapsids. The pellet was taken into 5 ml TMS buffer and applied to a 10–40% sucrose gradient in the same buffer. The sample was spun (130,000×*g*) for 3 h, at which point the procapsid band was visualized in ambient light. The band was harvested by aspiration and dialyzed against TMB buffer (50 mM Tris–HCl, pH 8.0, 15 mM MgCl_2_, 7 mM β-ME). The dialyzed procapsids were loaded onto a 50 ml DEAE-sepharose column equilibrated with TMB and the column was developed with a 0–250 mM NaCl gradient. The procapsid-containing fractions (50 mM NaCl) were pooled, dialyzed against TMB buffer, concentrated using an Ultrafree-15® filtration device (Millipore), and stored at 4 °C.

### Preparation of DNA substrate

A 13,881 kbp plasmid containing the *cos* site for packaging initiation, named pJM1, was prepared by the Feiss Lab (University of Iowa) and transformed into an *E. coli* strain. The plasmid was extracted with QIAGEN Plasmid Mini Kit. This plasmid was linearized and replicated by PCR using biotin and digoxigenin labeled primers, resulting in a ~ 13.7 kbp linear DNA with the *cos* site 10.1 kbp from the biotin labeled end for use in optical tweezers experiments. Post *cos* cleavage, there is a 10.1 kbp section of packageable DNA.

### Optical tweezers measurements

Our dual optical tweezers instrument was calibrated by our established method of using DNA molecules as metrology standards^38^. The efficient collection of lambda packaging data utilizes a protocol in which complexes are “pre-stalled” in a bulk reaction and then restarted in the optical tweezers instrument. Briefly, phage lambda procapsid-motor-DNA complexes are prepared in a solution consisting of 25 mM Tris–HCl pH 7.5 and 5 mM MgCl_2_ by mixing 17 nM lambda procapsids with 250 nM purified terminase and 11 ng of pJM1 DNA. In experiments where it was added, 50 nM of IHF is also added at this step. The mixture is left at room temperature for 5 min before 0.5 mM of ATP is added to initiate packaging. After another 45 s incubation period, 0.5 mM of γ-S-ATP is added in a final reaction volume of 5 μl which causes DNA packaging to stall^22^. The exposed end of the pJM1 DNA is biotinylated, allowing the stalled complexes to be attached to 2.1 μm diameter streptavidin coated microspheres (Spherotech). 0.5 μl of stalled complexes are added to 5 μl of SA (streptavidin) microspheres (5% w/v) and then incubated on a rotating incubator at room temperature for 20 min. Separately, 4 μl of lambda antibodies are mixed with 8 μl (5% w/v) of 2.3 μm diameter protein-G microspheres (Spherotech) and incubated on a rotating incubator at room temperature for 30 min to produce antibody-coated microspheres. The main flow cell buffer in which measurements are carried out consists of 25 mM Tris–HCl pH 7.5, 5 mM MgCl_2_, and 0.05 g L^−1^ BSA. 1 mM ATP is added to measure packaging. 

**Fig. 2:**
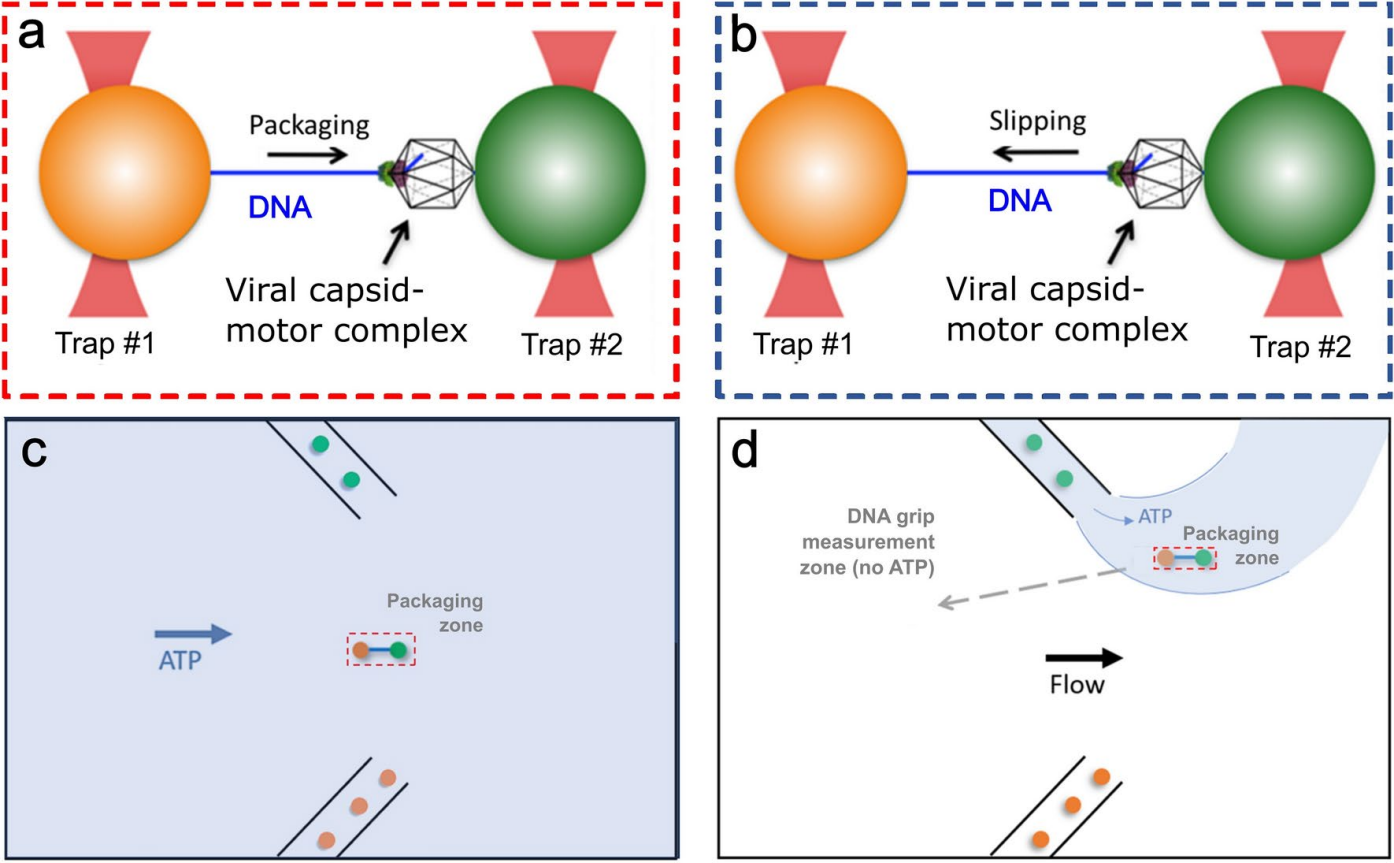
Schematic illustration of the optical tweezers measurements. Single molecule (**a**) packaging and (**b**) slipping measurement method. Packaging complexes stalled with γ-S-ATP are attached by the DNA end to a microsphere (orange) and trapped with optical tweezers. This microsphere is brought near a second trapped microsphere coated with antibodies that bind the capsid (green). The complex is then exposed to ATP in either (**c**) the main flow channel, or (**d**) a packaging zone near one of the inlet capillaries, such that the motor begins packaging the DNA. For the motor-DNA interaction measurements in the absence of nucleotide, after the complex packages ~ 6 kbp of DNA in the reaction solution with ATP it is moved (dashed arrow) into an upstream region of the fluid chamber to achieve rapid solution exchange.

The method of using these microsphere pairs to collect both packaging and slipping data with our dual trap system is illustrated and described in Fig. 2. Our general protocol was to wait at least several minutes after a DNA tethering event was observed to check for packaging activity, although the time was sometimes shorter because sometimes the DNA tethers spontaneously detach from the microspheres. The average time for packaging to initiate when IHF was included was 57 s (standard deviation 78 s) and in the experiments without IHF we waited longer (an average of 167 s, standard deviation 156 s) to give sufficient time to judge if packaging occurred (and in ~ 20% of trials we waited ~ 5–8 min).

### Data processing

Sections of data containing packaging (positive velocity) vs. pauses (zero velocity) vs. slips (negative velocity) were identified by setting velocity thresholds*.* We establish the value of the thresholds considering the inherent measurement noise measured in a control experiment in which DNA molecules alone were tethered between two microspheres to determine the uncertainty in velocity measurements for a static tether. For a 1 s detection window we measure an average velocity close to zero (0.004 bp/s), but with a standard deviation (s) of 14.5 bp/s that characterizes the uncertainty due to Brownian fluctuations and instrument noise. The threshold velocity to identify a period of pausing was set to be 3σ = 43.5 bp/s. Anything outside of this window is labeled as either packaging or slipping, depending on the direction of movement.

## Results

### Single-molecule assay using purified lambda terminase proteins

**Fig. 3:**
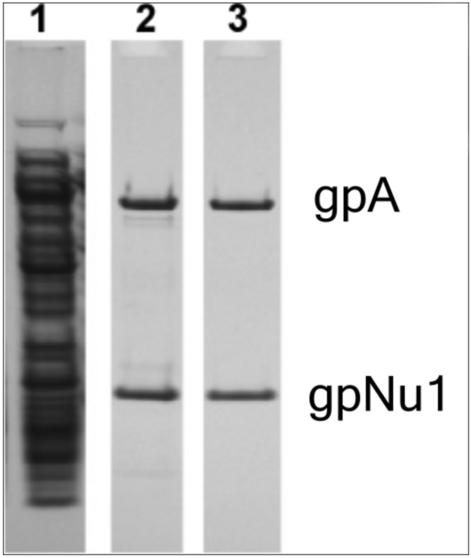
SDS-PAGE analysis of the terminase preparations utilized in this study. *Lane 1*, Terminase-containing crude cell extract. *Lane 2*, “Pure 1” purified terminase holoenzyme, *Lane 3*, “Pure 2” further purified terminase holoenzyme.

His-tagged lambda terminase holoenzyme was expressed in *E. coli* and crude cell extracts were prepared as described in Materials and Methods. The enzyme was then purified using Ni-chromatography (termed the “Pure 1” terminase) followed by Q-sepharose chromatography (termed the “Pure 2” terminase sample), respectively. SDS-PAGE analysis of the crude extract and purified preparations are shown in Fig. 3, which clearly demonstrates that the extract contains many additional host proteins.

For the optical tweezers measurements (see Materials and Methods for further details) we first mix purified terminase holoenzyme with purified lambda procapsids, a biotin end labeled DNA construct that contains a lambda *cos* site, and IHF protein (except in some experiments where it was omitted), in a buffer solution with ATP, to afford formation of a terminase-DNA complex which cleaves the DNA at the *cos* site (maturation complex, Fig. 1). This DNA substrate has a 10.1 kbp section between the biotin label and the *cos* site that we expect to be packaged. Post cleavage, the ring-shaped terminase complex attaches to the portal nanochannel ring on the procapsid to assemble the motor complex and packaging initiates (Fig. 1)^39^. After allowing a small amount of packaging to occur, we add excess γ-S-ATP, an ATP analog that halts packaging^22^ This affords a “stalled complex” composed of terminase bound to the portal of a partially filled shell and with the biotin-labeled end of the DNA hanging out of the procapsids. The motors are later restarted, allowing us to measure the remaining packaging with the optical tweezers.

The method for measuring packaging is illustrated schematically in Fig. 2. In brief, the stalled complexes assembled above are bound via the biotin labeled DNA end to streptavidin coated microspheres and injected into a fluid chamber via a micro-capillary. Microspheres coated with anti-lambda-procapsid antibodies are injected via a second capillary and one is trapped with an optical tweezers beam. A microsphere carrying the stalled complex is then trapped in a second beam and the two microspheres are moved into near contact. We then probe for a DNA molecule to become tethered between the two microspheres via binding of the procapsid to the antibody coated microsphere. The two microspheres are moved apart and, if a DNA molecule is tethered between them, this results in measured force on the microspheres. The fluid chamber contains a buffer solution with ATP which is gently flowing past the microspheres so that γ-S-ATP can dissociate from the stalled complexes and be replaced by ATP to restart packaging. We detect packaging by using a feedback control system that moves one microsphere relative to the other in order to maintain constant force. When packaging occurs, this results in the two microspheres moving closer together as the motor reels the DNA into the procapsid.

### Detection of DNA packaging events and dependence on IHF

**Fig. 4:**
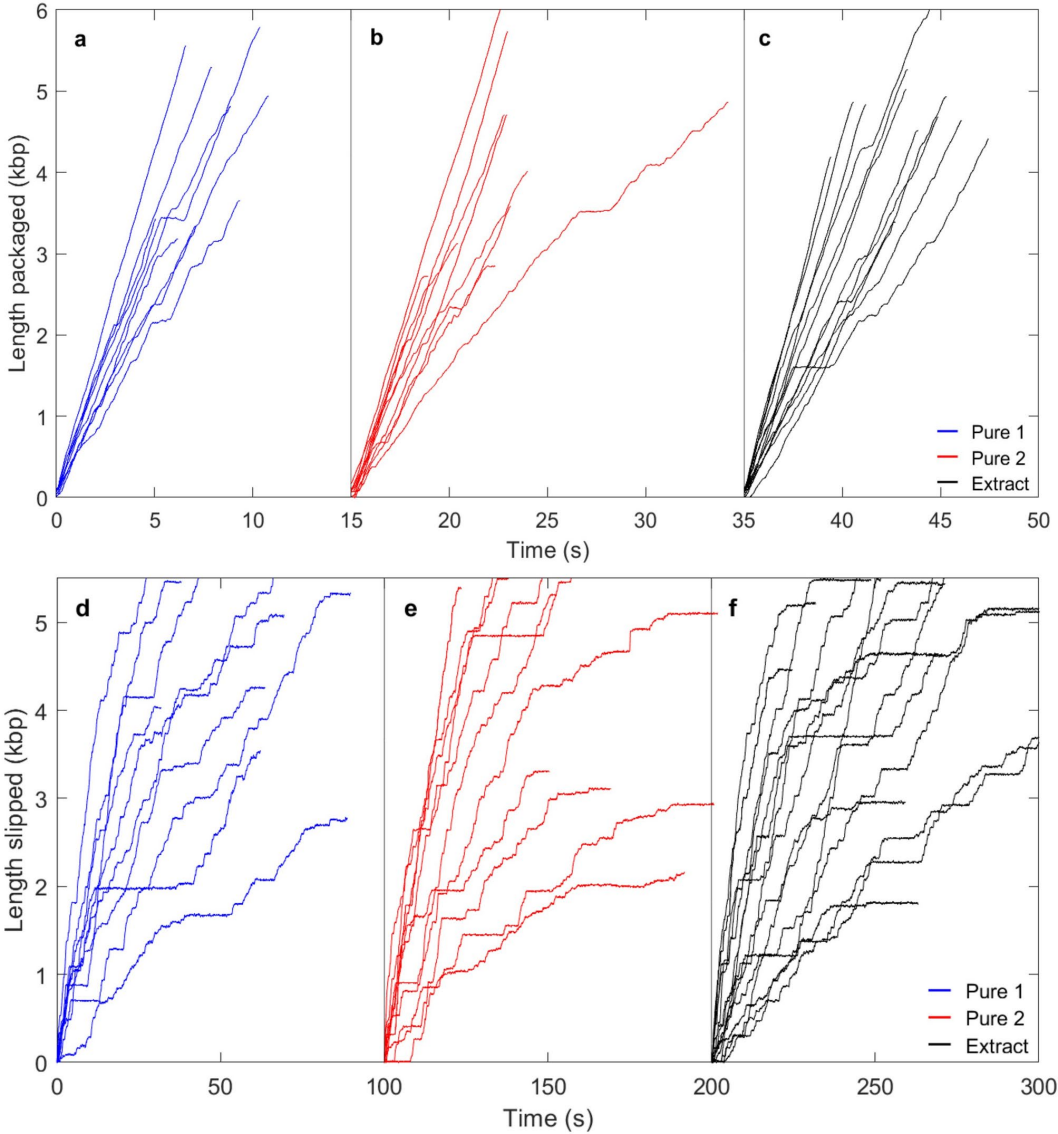
Examples of measurements of DNA packaging and slipping (aligned at length = 0 kbp). Packaging is in solution with 0.5 mM ATP (**a**–**c**; aligned at times 0, 15, and 35 s) and motor slipping/gripping in the absence of ATP (panels **d**–**f**; aligned at times 0, 100, and 200 s). Each plotted line is an event measured on a single complex. All measurements are with 5 pN applied force. The panel letters and line colors indicate complexes assembled with: (**a**,**d**) “Pure 1 + IHF” terminase (blue), (**b**,**e**) “Pure 2 + IHF” terminase (red), and (**c**,**f**) unpurified cell extract terminase (black).

Using the protocols described above we observed efficient detection of packaging events using both the “Pure 1 + IHF” and “Pure 2 + IHF” terminase samples (Fig. 4a,b). We quantified “packaging detection efficiency” as the number of measured packaging events per tethering event. For the “Pure 1 + IHF” and “Pure 2 + IHF” samples the efficiencies were 43% and 40%, respectively. In contrast, in the absence of IHF, we recorded 30 *tethering* events with each purified terminase but zero *packaging* events despite waiting, on average, a longer time than with IHF containing samples (see methods). For comparison, the measured packaging initiation efficiency when using the crude extract was 38%. Thus, with the described protocols we can now measure packaging with efficiency comparable to that in our prior studies that used crude terminase preparations. We note that the two different levels of purification did not result in significant differences, indicating there is no significant degradation of activity caused by extended purification.

To further characterize the packaging reaction, we compared the “initiation time”, defined as the measured time interval between detection of a DNA tether and detection of packaging. This was highly variable for individual complexes, regardless of terminase sample used, ranging from ~ 10–100 s. During this time the tether length remained relatively constant with only small fluctuations attributable to noise (examples of length vs. time measurements and further discussion is given in the Supplementary information). The average initiation time was ~ 30 s (SD ~ 40 s) for the measurements with crude terminase vs. ~ 60 s (SD ~ 80 s) with the purified terminase (Table 1). However, analysis of the time distributions showed that this difference is not statistically significant (Supplementary Fig. S1). Thus, our data suggest that additional substances in the crude extract do not make any difference.

**Table 1:** Averaged metrics characterizing the packaging dynamics.

Packaging Metrics—Mean values (SD) [SEM]						
Sample	Packaging rate (bp/s)	Motor velocity (bp/s)	Pause frequency (#/kbp)	Initiation time (s)	Pause duration (s)	Packaging detection efficiency
Extract (25)	460 (291) [6.3]	514 (264) [6.1]	0.37 (1.1) [0.22]	31 (39) [6.7]	0.55 (0.9) [0.14]	38%
Pure 1 (30)	n/a	n/a	n/a	n/a	n/a	0%
Pure 1 + IHF (13)	477 (198) [6.4]	510 (199) [6.2]	0.28 (0.45) [0.12]	59 (69) [21]	0.25 (0.28) [0.07]	43%
Pure 2 (30)	n/a	n/a	n/a	n/a	n/a	0%
Pure 2 + IHF (23)	470 (240) [7.1]	504 (221) [6.8]	0.43 (0.5) [0.13]	55 (91) [23]	0.40 (0.41) [0.08]	40%

Measurements were taken with 0.5 mM ATP and 5 pN applied force using the indicated terminase samples. The number in parentheses next to the sample name indicates the number of recorded packaging events (or number of tethers in the experiments without IHF). Packaging rate refers to the DNA translocation velocity including pauses whereas the motor velocity excludes pauses. The standard deviation in the values is indicated in parentheses “()”, and the standard error in the mean is indicated in brackets “[]”. Packaging rate and motor velocity are averaged over 1 s time intervals and the pause frequency and initiation time are averaged over individual packaging events. No packaging was measured for the “Pure 1” and “Pure 2” samples without IHF.

An interesting feature observed when using purified terminase with no IHF is that tethering of the DNA was consistently detected, again with the tether length being nearly constant in time (see Supplementary Fig. S2), but the lengths of the individual tethers varied much more widely than those observed in the experiments with IHF (Supplementary Fig. S2), ranging from significantly shorter to significantly longer. Notably, some tethers were longer than expected if terminase were binding at the *cos* site, suggesting that the DNA had not been cleaved at the *cos* site (see additional discussion in the Supplementary information). These data suggest that complexes assembled in the absence of IHF bind the DNA non-specifically at a range of positions upstream and downstream of the *cos* site, including the section upstream of the *cos* site that would have been cleaved away if terminase cleaved the DNA at the *cos* site. These findings are consistent with ensemble biochemical studies that have demonstrated that terminase binds *cos*-containing DNA duplexes non-specifically in the absence of IHF and that duplex nicking at *cos* requires IHF^37,40^.

### Packaging dynamics and motor force

To evaluate whether contents of the *E. coli* extract, besides IHF, have any effects on the motor function, we compared optical tweezers measurements made with the crude terminase extract vs. those made using the “pure 1” and “pure 2” terminase samples, in the presence of IHF. Examples of individual DNA length packaged vs. time events, measured with 0.5 mM ATP and 5 pN applied force, are shown in Fig. 4a–c, illustrating similar results for each sample. The ATP concentration of 0.5 mM was chosen because it was found in previous studies to be saturating, *i.e.* to allow the motor to translocate at maximum speed^41,42^. A 5 pN applied force is used because it keeps the unpackaged DNA segment sufficiently stretched so its length can be accurately measured, but it is small compared to the maximum force the motor can exert, so causes only minor perturbation of the motor function. We further analyzed these datasets to determine several averaged metrics, presented in Table 1.

**Fig. 5:**
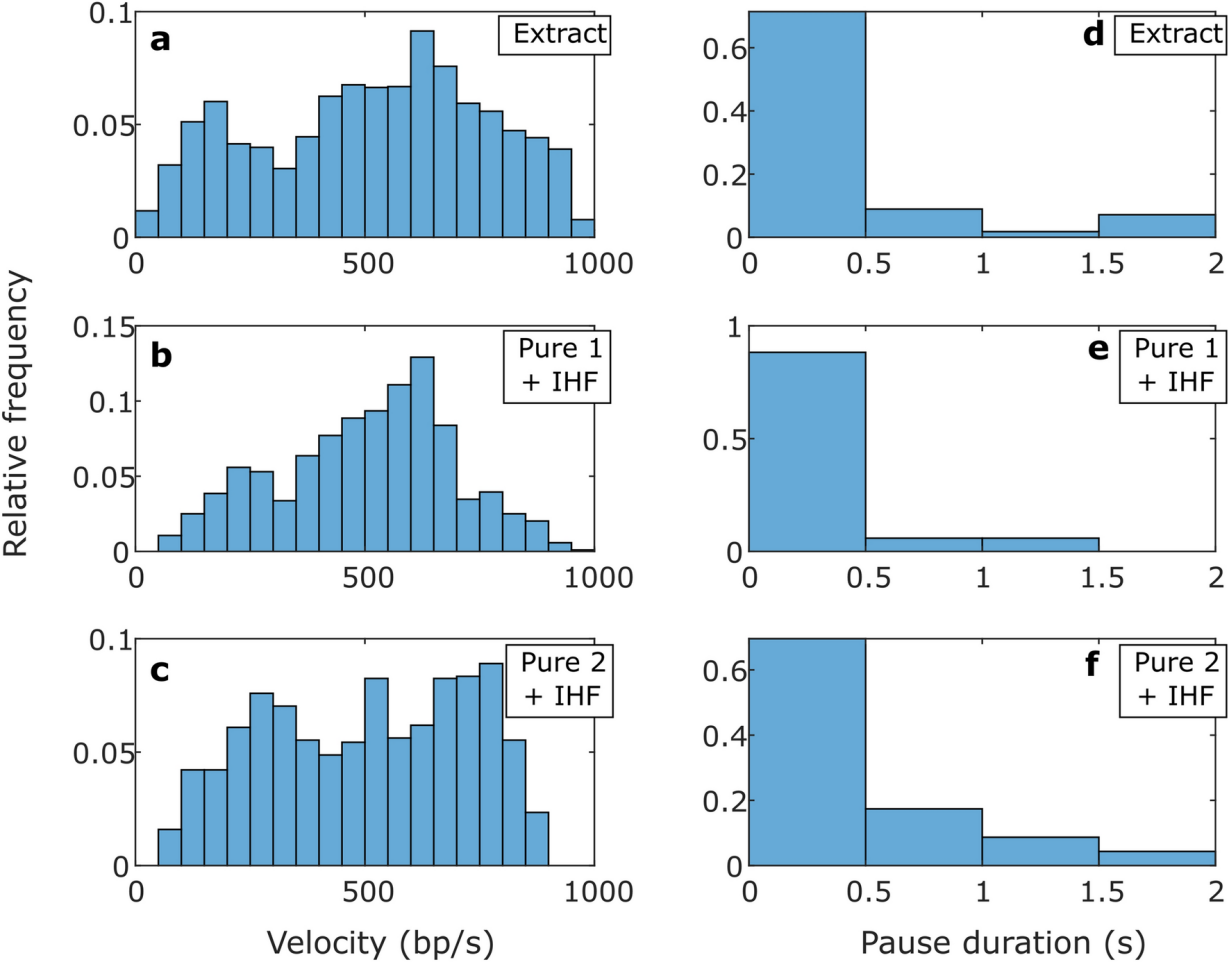
Packaging statistics. (**a**–**c**) Distributions of motor velocities measured in 1 s time intervals with 0.5 mM ATP and 5 pN applied force, with the samples indicated in the legends. (**d**–**f**) Distribution of pause durations during packaging, with the samples indicated in the legends. Panels (**a**,**d**) are measurements with the unpurified cell extract terminase, panels (**b**,**e**) are with the “Pure 1 + IHF” terminase, and panels (**c**,**f**) are with the “Pure 2 + IHF” terminase. The number of data points that went into the distributions shown in plots a-f are 1875, 41, 1038, 17, 1067, and 23, respectively.

Three key metrics of packaging dynamics are the “average motor velocity”, which refers to the average DNA translocation velocity not including pauses, and the frequency and average duration of pauses. No significant differences were observed in these parameters for the crude vs. purified terminase samples (Table 1), *i.e.* any minor differences were small compared with the standard deviations in the quantities. For example, the average motor velocity is around 500 bp/s for each, with differences of only ~ 10 bp/s, which is small compared with the ~ 200 bp/s standard deviations in the velocities measured. We also plot the distributions of transient velocities in Fig. 5a–c, and the distributions of pause durations in Fig. 5d–f, revealing that they are essentially indistinguishable for the different samples.

Another important metric of motor function is the force generating capability. In previous studies with crude terminase preparations, we measured a maximum motor force of ~ 50 pN. This figure is a lower bound because applying this level of force induces rapid detachment of the tethered DNA, attributable to dissociation of the antibody-procapsid bond. This bound came from measurements in which the force was rapidly ramped during packaging and translocation events were detected prior to dissociation^19^. Here, we used the same approach to test packaging complexes assembled using the purified terminase and similarly found that the motor was capable of generating at least 50 pN of force.

### Nucleotide independent motor-DNA interactions

We recently developed a method to characterize motor-DNA interactions in the absence of ATP and apply this method here to further investigate if there are any differences in the motor complexes assembled using purified terminase^21,22^. In this approach, we suddenly move a complex packaging DNA in a buffer with ATP into a region of the fluid chamber without ATP. This causes DNA translocation to abruptly stop, and the DNA slips out of the capsid. Importantly, we observe a slow transient slipping velocity which indicates significant friction between the motor and DNA, and frequent interruption of the slipping by pauses, which are events where the motor transiently grips the DNA.

**Fig. 6:**
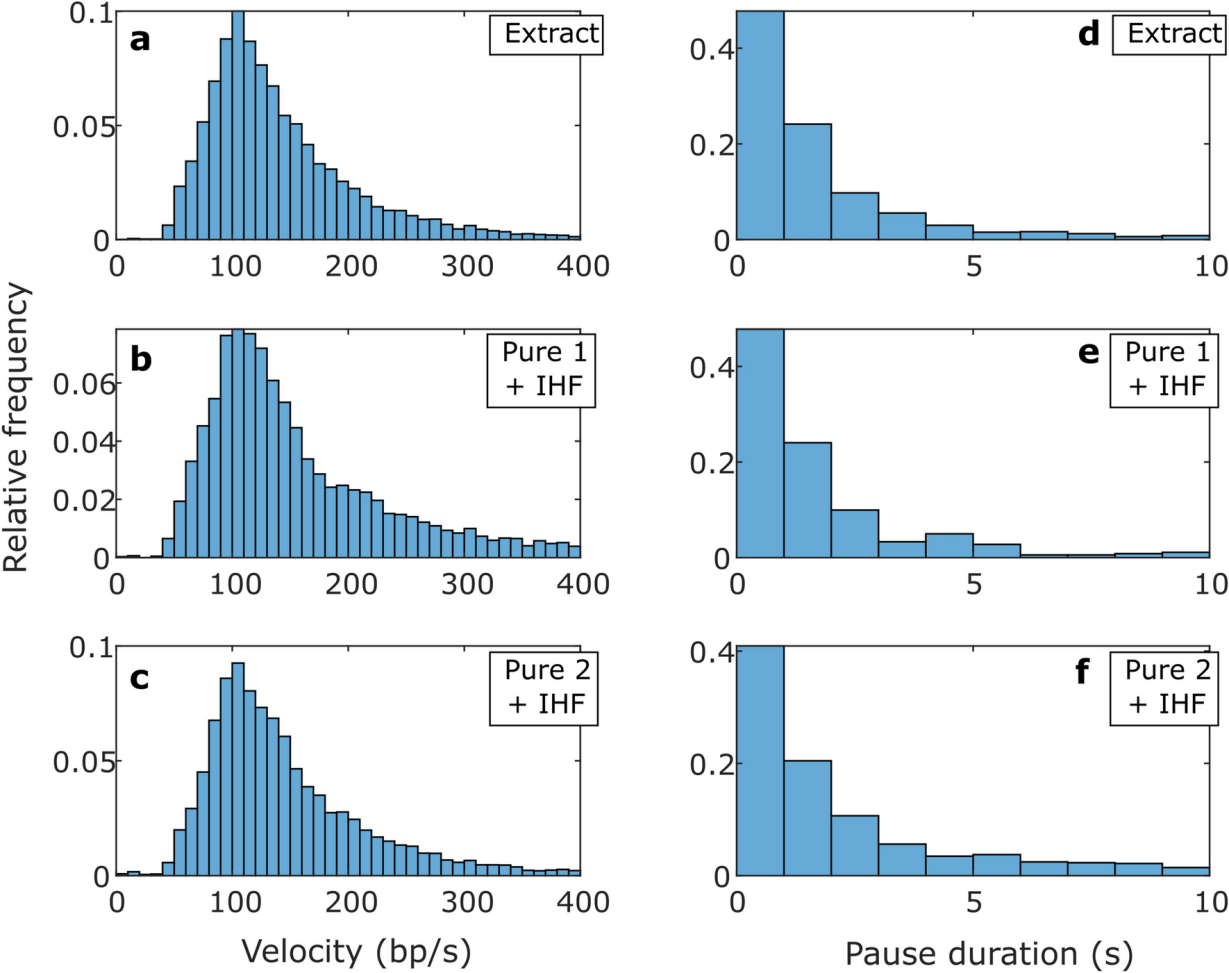
Slipping statistics. (**a**–**c**) Distributions of transient slip velocities measured in 1 s time intervals with no ATP and 5 pN applied force, with the samples indicated in the legends. (**d**–**f**) Distribution of pause durations during slipping (motor-DNA gripping events), with the samples indicated in the legends. Panels (**a**,**d**) are measurements with the unpurified cell extract terminase, panels (**b**,**e**) are with the “Pure 1 + IHF” terminase, and panels (**c**,**f**) are with the “Pure 2 + IHF” terminase. The number of data points that went into the distributions shown in plots a-f are 16631, 974, 5981, 355, 5032, and 275, respectively.

Measurements of the length of DNA that has slipped out vs. time are shown in Fig. 4d–f, showing similar results for the three different samples. We analyzed these datasets to determine averaged metrics characterizing the dynamics: overall DNA exit velocity (including pauses), transient slip velocity (excluding pauses), % time slipping, pause frequency, and pause and slip duration. As shown in Table 2, we find no significant differences for the crude vs. purified terminase samples with IHF, *i.e.* any minor differences were small compared with the standard deviations. For example, the average transient slip velocity is around 150 bp/s in each case, differing by only ~ 10 bp/s for different samples, which is small compared with the standard deviations of ~ 80 bp/s. We also plot the distributions of transient slipping velocities (Fig. 6a–c) and the distributions of pause durations during slipping (lifetime of individual motor-DNA gripping events; Fig. 6d–f) and find that they are essentially indistinguishable for the different samples.

**Table 2:** Averaged metrics characterizing the slipping motor-DNA interactions (slips and gripping events).

Slipping metrics—Mean values (SD) [SEM]						
Sample	DNA exit velocity (bp/s)	Transient slip velocity (bp/s)	% Time slipping	Pause frequency (#/kbp)	Pause duration (s)	Slip duration (s)
Extract (53)	60 (77) [0.39]	148 (70) [0.54]	51% (18) [2.5]	4.3 (2.5) [0.35]	2.3 (4.1) [0.13]	1.7 (1.8) [0.06]
Pure 1 + IHF (19)	61 (91) [0.73]	159 (91) [1.2]	53% (22) [5]	4 (2.9) [0.68]	2.7 (6.2) [0.32]	1.6 (1.8) [0.1]
Pure 2 + IHF (22)	60 (85) [0.7]	154 (80) [1.1]	50% (24) [5.2]	4.3 (2.2) [0.45]	2.9 (5.4) [0.33]	1.8 (1.9) [0.1]

Measurements were taken with no ATP and 5 pN applied force with the indicated terminase samples. The number in parentheses next to the sample name indicates the number of recorded DNA release events. DNA exit velocity refers to the average velocity including pauses whereas transient slip velocity excludes pauses. The standard deviation in the values is indicated in parentheses “()”, and the standard error in the mean is indicated in brackets “[]”. The exit velocity and transient slip velocity are averaged over 1 s time intervals while the % time slipping and pause frequency are averaged over individual DNA release events.

## Discussion and conclusions

A major result of this study is the development of methods for *efficient* measurement of phage lambda DNA packaging with optical tweezers using a defined, minimal system of purified components: procapsids, DNA, terminase holoenzyme (TerL·TerS_2_), IHF, and ATP. The efficiency of detecting packaging events is as high as in prior studies which used crude cell extracts containing terminase. The defined system will be advantageous for future studies to investigate effects of other phage proteins on the packaging dynamics.

Our finding that *E. coli* IHF protein is required to measure lambda packaging in the optical tweezers assay is consistent with the findings of previous ensemble biochemical studies which found that IHF strongly stimulates *cos* cleavage activity, which is an essential step in the initiation of packaging^37^. In bulk assays, DNA packaging and viral assembly activities were detected in the absence of IHF, but only at a very low level. This is consistent with our findings since the optical tweezers assay probes individual complexes one-by-one, making high activity essential. Our data provide evidence that, in the absence of IHF, terminase tends to non-specifically bind to the DNA rather than to the *cos* site (Supplementary Fig. S2), which is consistent with biochemical data^40^. This suggests that (i) terminase binds DNA non-specifically in the absence of IHF and that (ii) IHF is required for site-specific assembly of terminase at the *cos* site.

Another major finding is that there are no significant differences in multiple metrics of motor function, including details of the packaging dynamics, motor force generation, and nucleotide-independent motor-DNA interactions, with the crude vs. purified terminase preparations. Although it is preferable for future studies to use purified samples, these findings indicate that our prior studies that used unpurified samples likely did not suffer any undesirable artifacts due to components in the crude cell extracts. More generally, the findings suggest that no host cell components in the extracts, besides IHF, significantly affect the assembly of the motor complex or its conformation/state with regards to the initiation of genome packaging and function during DNA translocation.

We emphasize that our findings provide information only about the initial stages of packaging because we are measuring only packaging of ~ 15% of the full lambda genome length. Other proteins may play a role in facilitating packaging of the full 48.5 kbp lambda genome. Indeed, for example, our previous studies suggest that an accessory capsid protein, gpD, is needed to maintain capsid stability to package the full genome^19,43^. The assay described here, using a defined set of purified proteins, sets the stage for rigorous characterization of additional phage lambda proteins that are thought to be important in downstream packaging events, such as gpFI, gpW, gpFII and perhaps even phage tails, using single molecule approaches that will strongly complement ensemble biochemical studies.

## Supplementary Information

Supplementary Information.

## Data Availability

The datasets plotted in the paper are available from the first author on reasonable request.

## References

[1] Casjens, S. R. The DNA-packaging nanomotor of tailed bacteriophages. *Nat. Rev. Microbiol. ***9**, 647–657 (2011). 21836625 10.1038/nrmicro2632

[2] Hetherington, C., Moffitt, J., Jardine, P. & Bustamante, C. *Comprehensive Biophysics* (Elsevier Inc, 2012).

[3] Roizman, B. Herpes simplex viruses. In *Fields Virology* 2501–2602 (Lippincott Williams & Wilkins, 2007).

[4] Jardine, P. J. & Anderson, D. L. DNA packaging in double-stranded DNA phages. *The Bacteriophages ***2**, 49–65 (2006).

[5] Catalano, C. E. & Morais, M. C. Viral genome packaging machines: Structure and enzymology. *The Enzymes ***50**, 369–413 (2021). 34861943 10.1016/bs.enz.2021.09.006

[6] Rao, V. B. & Feiss, M. Mechanisms of DNA packaging by large double-stranded DNA viruses. *Annu. Rev. Virol. ***2**, 351–378 (2015). 26958920 10.1146/annurev-virology-100114-055212 PMC4785836

[7] Riemer, S. C. & Bloomfield, V. A. Packaging of DNA in bacteriophage heads: Some considerations on energetics. *Biopolymers ***17**, 785–794 (1978). 638234 10.1002/bip.1978.360170317

[8] Smith, D. E. et al. The bacteriophage φ29 portal motor can package DNA against a large internal force. *Nature ***413**, 748–752 (2001). 11607035 10.1038/35099581

[9] Evilevitch, A., Lavelle, L., Knobler, C. M., Raspaud, E. & Gelbart, W. M. Osmotic pressure inhibition of DNA ejection from phage. *Proc. Natl. Acad. Sci. USA ***100**, 9292–9295 (2003). 12881484 10.1073/pnas.1233721100 PMC170911

[10] Harvey, S. C., Petrov, A. S., Devkota, B. & Boz, M. B. Viral assembly: A molecular modeling perspective. *Phys. Chem. Chem. Phys. ***11**, 10553–10564 (2009). 20145801 10.1039/b912884k

[11] Baines, J. D. Herpes simplex virus capsid assembly and DNA packaging: A present and future antiviral drug target. *Trends Microbiol. ***19**, 606–613 (2011). 22000206 10.1016/j.tim.2011.09.001

[12] Hayes, J. A. & Kelch, B. A. In *Encyclopedia of Virology* 4th edn (eds Bamford, D. H. & Zuckerman, M.) 148–159 (Academic Press, 2021).

[13] Rao, V. B. & Feiss, M. The bacteriophage DNA packaging motor. *Annu. Rev. Genet. ***42**, 647–681 (2008). 18687036 10.1146/annurev.genet.42.110807.091545

[14] Chen, W. et al. Structural changes of a bacteriophage upon DNA packaging and maturation. *Protein Cell ***11**, 374–379 (2020). 32266588 10.1007/s13238-020-00715-9 PMC7196576

[15] Fung, H. K. et al. Structural basis of DNA packaging by a ring-type ATPase from an archetypal viral system. *Nucleic Acids Res. ***50**, 8719–8732 (2022). 35947691 10.1093/nar/gkac647 PMC9410871

[16] Bayfield, O. W. et al. Cryo-EM structure and in vitro DNA packaging of a thermophilic virus with supersized T= 7 capsids. *Proc. Natl. Acad. Sci. USA ***116**, 3556–3561 (2019). 30737287 10.1073/pnas.1813204116 PMC6397560

[17] Keller, N., del Toro, D. J. & Smith, D. E. Single-molecule measurements of motor-driven viral DNA packaging in bacteriophages Phi29, Lambda, and T4 with optical tweezers. *Molecular Motors: Methods and Protocols* 393–422 (2018). 10.1007/978-1-4939-8556-2_20 29971729

[18] Smith, D. E. Single-molecule studies of viral DNA packaging. *Curr. Opin. Virol. ***1**, 134–141 (2011). 22440623 10.1016/j.coviro.2011.05.023 PMC4393007

[19] Fuller, D. N. et al. Measurements of single DNA molecule packaging dynamics in bacteriophage λ reveal high forces, high motor processivity, and capsid transformations. *J. Mol. Biol. ***373**, 1113–1122 (2007). 17919653 10.1016/j.jmb.2007.09.011 PMC3311920

[20] Fuller, D. N., Raymer, D. M., Kottadiel, V. I., Rao, V. B. & Smith, D. E. Single phage T4 DNA packaging motors exhibit large force generation, high velocity, and dynamic variability. *Proc. Natl. Acad. Sci. USA ***104**, 16868–16873 (2007). 17942694 10.1073/pnas.0704008104 PMC2040459

[21] Ordyan, M., Alam, I., Mahalingam, M., Rao, V. B. & Smith, D. E. Nucleotide-dependent DNA gripping and an end-clamp mechanism regulate the bacteriophage T4 viral packaging motor. *Nat. Commun. ***9**, 5434 (2018). 30575768 10.1038/s41467-018-07834-2 PMC6303390

[22] Rawson, B. *et al*. Regulation of phage lambda packaging motor-DNA interactions: Nucleotide independent and dependent gripping and friction. *bioRxiv* 2022–2009 (2022).

[23] Grimes, S., Jardine, P. J. & Anderson, D. *Bacteriophage φ29 DNA Packaging* (2002). 10.1016/s0065-3527(02)58007-6 12205781

[24] Catalano, C. E., Feiss, M. & Catalano, C. E. Bacteriophage lambda terminase and the mechanism of viral DNA packaging. *Viral Genome Packaging Machines: Genetics, Structure, and Mechanism* 5–39 (2005).

[25] Kondabagil, K. R., Zhang, Z. & Rao, V. B. The DNA translocating ATPase of bacteriophage T4 packaging motor. *J. Mole. Biol. ***363**, 786–799 (2006). 10.1016/j.jmb.2006.08.054 16987527

[26] Black, L. W. & Peng, G. Mechanistic coupling of bacteriophage T4 DNA packaging to components of the replication-dependent late transcription machinery. *J. Biol. Chem. ***281**, 25635–25643 (2006). 16807240 10.1074/jbc.M602093200

[27] Gaussier, H., Yang, Q. & Catalano, C. E. Building a virus from scratch: Assembly of an infectious virus using purified components in a rigorously defined biochemical assay system. *J. Mol. Biol. ***357**, 1154–1166 (2006). 16476446 10.1016/j.jmb.2006.01.013

[28] Prokhorov, N. S. et al. Biophysical and structural characterization of a multifunctional viral genome packaging motor. *Nucleic Acids Res. ***52**, 831–843 (2024). 38084901 10.1093/nar/gkad1135 PMC10810279

[29] Yang, Q. & Catalano, C. E. ATP serves as a nucleotide switch coupling the genome maturation and packaging motor complexes of a virus assembly machine. *Nucleic Acids Res. ***48**, 5006–5015 (2020). 32255177 10.1093/nar/gkaa205 PMC7229814

[30] Ortiz, D. et al. Walker-A motif acts to coordinate ATP hydrolysis with motor output in viral DNA packaging. *J. Mol. Biol. ***428**, 2709–2729 (2016). 27139643 10.1016/j.jmb.2016.04.029 PMC4905814

[31] Davidson, A. & Gold, M. A novel in vitro DNA packaging system demonstrating a direct role for the bacteriophage λ FI gene product. *Virology ***161**, 305–314 (1987). 2961121 10.1016/0042-6822(87)90122-x

[32] Yang, Q. & Catalano, C. E. Biochemical characterization of bacteriophage lambda genome packaging in vitro. *Virology ***305**, 276–287 (2003). 12573573 10.1006/viro.2002.1602

[33] Gaussier, H., Ortega, M. E., Maluf, N. K. & Catalano, C. E. Nucleotides regulate the conformational state of the small terminase subunit from bacteriophage lambda: Implications for the assembly of a viral genome-packaging motor. *Biochemistry ***44**, 9645–9656 (2005). 16008350 10.1021/bi050333e

[34] Kornberg, A. *Methods in Enzymology* 3–6 (Elsevier, 2009). 10.1016/S0076-6879(09)63001-9 19892161

[35] Ortiz, D. et al. Evidence that a catalytic glutamate and an ‘Arginine Toggle’ act in concert to mediate ATP hydrolysis and mechanochemical coupling in a viral DNA packaging motor. *Nucleic Acids Res. ***47**, 1404–1415 (2019). 30541105 10.1093/nar/gky1217 PMC6379665

[36] Ortiz, D. et al. Functional dissection of a viral DNA packaging Machine’s Walker B Motif. *J. Mol. Biol. ***431**, 4455–4474 (2019). 31473160 10.1016/j.jmb.2019.08.012 PMC7416571

[37] Chang, J. R., Andrews, B. T. & Catalano, C. E. Energy independent helicase activity of a viral genome packaging motor. *Biophys. J. ***102**, 642a (2012). 10.1021/bi201604b PMC3266165 22191393

[38] Rickgauer, J. P., Fuller, D. N. & Smith, D. E. DNA as a metrology standard for length and force measurements with optical tweezers. *Biophys. J. ***91**, 4253–4257 (2006). 16963512 10.1529/biophysj.106.089524 PMC1635671

[39] Maluf, N. K., Gaussier, H., Bogner, E., Feiss, M. & Catalano, C. E. Assembly of bacteriophage lambda terminase into a viral DNA maturation and packaging machine. *Biochemistry ***45**, 15259–15268 (2006). 17176048 10.1021/bi0615036

[40] Yang, T.-C. et al. Physical and functional characterization of a viral genome maturation complex. *Biophys. J. ***112**, 1551–1560 (2017). 28445747 10.1016/j.bpj.2017.02.041 PMC5406279

[41] Tomka, M. A. & Catalano, C. E. Kinetic characterization of the ATPase activity of the DNA packaging enzyme from bacteriophage. *Biochemistry ***32**, 11992–11997 (1993). 8218275 10.1021/bi00096a008

[42] Woods, L. & Catalano, C. E. Kinetic characterization of the GTPase activity of phage λ terminase: Evidence for communication between the two “NTPase” catalytic sites of the enzyme. *Biochemistry ***38**, 14624–14630 (1999). 10545186 10.1021/bi990866l

[43] Yang, Q., Maluf, N. K. & Catalano, C. E. Packaging of a unit-length viral genome: The role of nucleotides and the gpd decoration protein in stable nucleocapsid assembly in bacterio-phage λ. *J. Mol. Biol. ***383**, 1037–1048 (2008). 18801370 10.1016/j.jmb.2008.08.063

