# Supplementary material for: Single-molecule measurements of bacteriophage lambda DNA packaging using purified terminase motor proteins and E. coli integration host factor
Source: Sci Rep. 2025 Feb 27;15:7093. doi: 10.1038/s41598-024-74915-2 (PMC11868608; doi:10.1038/s41598-024-74915-2)
Supplement: Supplementary file 1 — Supplementary Information. [file 41598_2024_74915_MOESM1_ESM.docx]

**Supplemental Information for:**

**Single-Molecule Measurements of Bacteriophage Lambda DNA Packaging Using Purified Terminase Motor Proteins and *E. coli* Integration Host Factor**

Brandon Rawson^1^, Qin Yang^2^, Carlos E. Catalano^2^, Douglas E. Smith^1^*


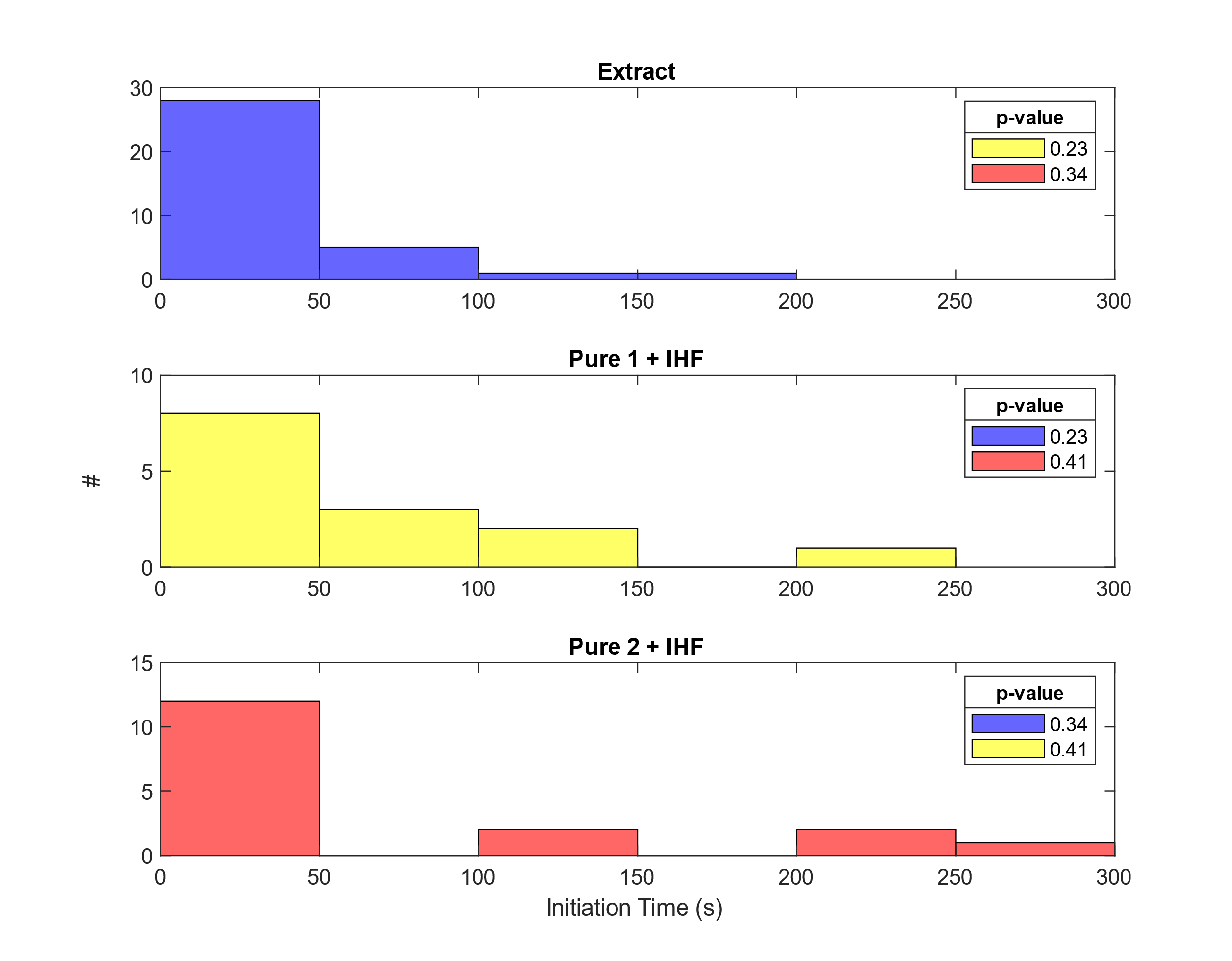


**Supplementary Figure S1.** Distributions of initiation times (i.e., the measured time between detection of a DNA tether and initiation of packaging), for complexes assembled with the crude extract and the purified terminase samples( pure 1 + IHF and pure 2 + IHF) (blue, yellow, and red, respectively). The ensembles of time values were also compared via two-sample K-S tests which did not reveal any statistically significant differences between them (p-values for each comparison are given in the legends). We also note that we do not find any significant correlation between initial tether length and initiation time: Spearman correlation coefficient = 0.178).


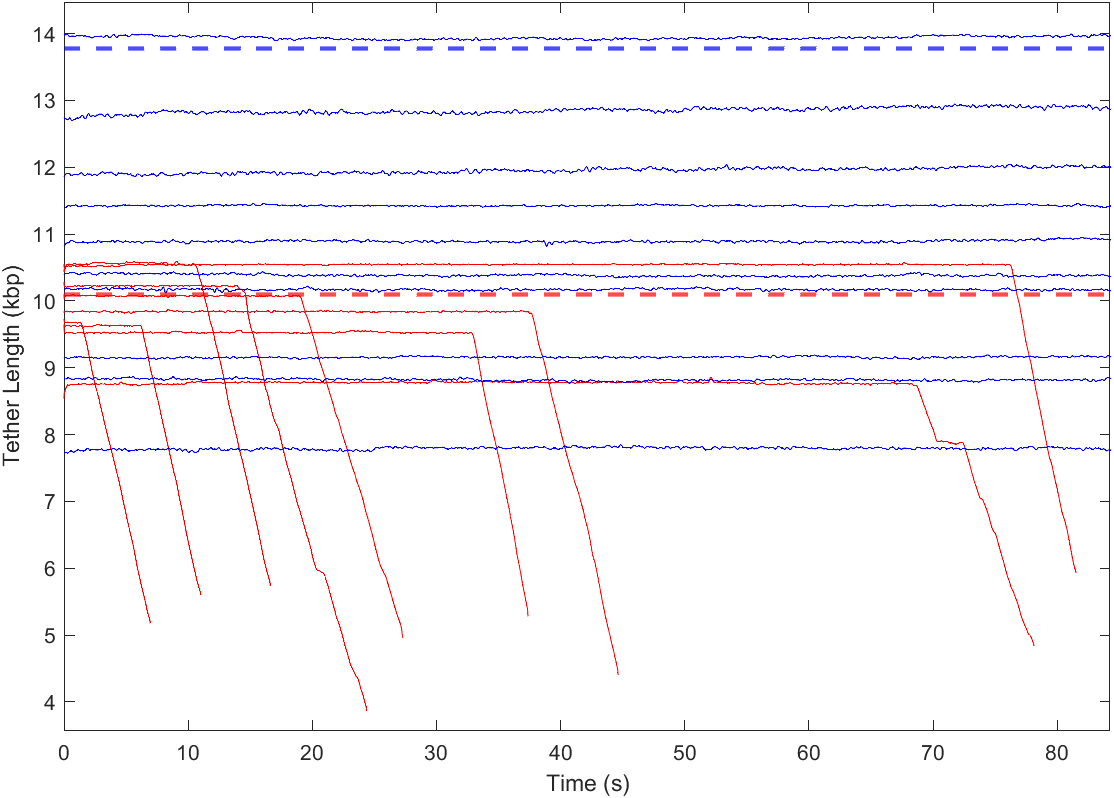


**Supplementary Figure S2.** Plots of tethers formed using purified components with (red) and without (blue) IHF. In the case of “with IHF”, the subsequent packaging portion of the trace is also shown. The dashed lines show the expected tether lengths of our DNA substrate, either cleaved at the cos site (red), or uncleaved (blue).

**Additional discussion:** Note that the initial tether lengths remain nearly constant. The small fluctuations seen in the plot are likely artifacts due to instrumental noise (e.g., mechanical disturbances or tiny particles of “dirt” moving into or out of the optical traps). In the measurements without IHF the overall level of fluctuations was larger (standard deviation of 65.4 bp vs. 38.7 bp for those with IHF), but because noise levels vary and the net tether shortenings are always less than the standard deviation of the fluctuations, we do not attribute these fluctuations to small packaging and slipping events. The differences are likely attributable to minor differences in instrumental noise during different measurement periods.

Another important point is that in the measurements with IHF, there are significant variations in the average initial tether lengths attributable to variations in the length of DNA packaged before complexes are stalled with γ-S-ATP. Additionally, in all measurements there are significant variations in tether length due to inaccuracy in the absolute measurement of individual DNA lengths due to microsphere size variations. Relative length changes are measured much more accurately than absolute values (see: Y. Mo et al, Front. Mol. Biosci. 8:605102, 2021).

However, considering these sources of variation, the DNA tether lengths measured in the experiments with IHF are consistent with packaging initiating at the *cos* site. In contrast, for the experiments without IHF, the variation in tether lengths is much wider, ranging from significantly shorter than, to significantly longer than those observed with IHF. That some of the lengths were longer than the 10.1 kbp length we expect if DNA was cleaved at the *cos* site and approaching the full ~13.7 kbp length of the uncleaved DNA suggests that the DNA is not cleaved in the absence of IHF and terminase is binding the DNA non-specifically at a range of positions both upstream and downstream of the cos site.
